# An essential role of PI3K in the control of West Nile virus infection

**DOI:** 10.1038/s41598-017-03912-5

**Published:** 2017-06-16

**Authors:** Leilei Wang, Long Yang, Erol Fikrig, Penghua Wang

**Affiliations:** 1 Department of Microbiology and Immunology, School of Medicine, New York Medical College, Valhalla, 10595 NY USA; 2 Department of Obstetrics and Gynecology, Shengjing Hospital of China Medical University, Shenyang, 110004 China; 3 Section of Infectious Diseases, Yale University School of Medicine, 300 Cedar St, New Haven, CT 06510 USA; 4 Howard Hughes Medical Institute, Chevy Chase, Maryland, USA

## Abstract

The phosphatidyl-inositol-3 kinases (PI3K) pathway regulates a variety of cellular processes, including cell proliferation, RNA processing, protein translation, autophagy, apoptosis and antiviral immunity. Many viruses depend on PI3K signaling for replication. However, its role in flaviviral infection has not been clearly defined. Here we report that PI3K signaling is critical for the control of West Nile virus (WNV) infection by regulating type I IFN (IFN-I) response. Inhibition of PI3K activity by 3-methyl adenine (3-MA), Wortmannin (WM) and LY294002 (LY) increased viral titers by 3–16 folds in primary mouse macrophages, embryonic fibroblasts and human cell lines. Both 3-MA and LY repressed IFN-I mRNA and protein expression significantly. Surprisingly, WM enhanced the mRNA expression of IFN-I and TNF-α, and TNF-α protein production modestly, while dramatically decreased the secreted IFN-I. Further studies showed that the catalytic subunit p110δ of class I PI3K played a role in induction of antiviral immune responses. Lastly translocation of interferon regulatory factor 7(IRF7) from the cytosol to the nuclei was effectively blocked in the presence of PI3K inhibitors. Our results clearly define an antiviral role of PI3K by modulating immune responses and demonstrate differential mode of action of three PI3K inhibitors on IFN-I.

## Introduction

WNV is an enveloped, single stranded, positive-sense RNA virus, belonging to the *Flaviviridae* family, which includes a number of medically important human pathogens of global epidemiological importance such as hepatitis C (HCV), dengue (DENV), Zika virus (ZIKV), yellow fever (YFV), Japanese encephalitis (JEV), St. Louis encephalitis (SLEV), and tick-borne encephalitis (TBEV) viruses. WNV has been more widely recognized since 1999 when it was first introduced into North America and was accountable for 7 deaths^1^. WNV can now be found all over the United States continent, and has been associated with over 21,000 encephalitis/meningitis cases and 1,800 deaths (https://www.cdc.gov/westnile/statsmaps/index.html). The mortality for the population over 70 years old can be as high as 15% to 29%^2^. Moreover 50% of those elderly who survived WNV infection may have significant post-illness morbidity for at least a year^3^. However, current therapeutic options for WNV disease are mainly supportive^4^, due to lack of effective vaccines and specific antiviral drugs.

Host control of WNV infection depends on the innate immune system during the acute phase and on the antibody response later^3, 5^. The phosphatidyl-inositol-3 kinases (PI3K)/ serine/threonine kinase (Akt) pathway regulates a variety of cellular processes, including cell proliferation, RNA processing, protein translation, autophagy and apoptosis^6–9^. It also plays an important role in induction of antiviral responses. For instance, vesicular stomatitis virus (VSV), Toll like receptor 3 (TLR3) and TLR4 agonists induced phosphorylation of IFN regulatory factor 3 (IRF3) via the PI3K/Akt pathway^10, 11^, and subsequent type I interferon (IFN-I) response, a critical antiviral mechanism. In addition, PI3K/Akt is essential for TLR3-mediated tyrosine phosphorylation, and RIG-I (RLR) dependent IRF3 activation in response to double/single –stranded RNA (ds/ss RNA) viruses^12–15^. On the other hand, PI3K/Akt signaling promotes cellular survival and autophagy^16^, another important cell-intrinsic antiviral mechanism^17^. Hepatitis B was reported to activate the PI3K pathway and thus inhibit TGFβ-induced apoptosis^15^. Another study showed that two faviviruses, JEV and DEN-2 activated the PI3K/Akt pathway and then blocked early apoptotic cell death^18^. The role of the PI3K/Akt pathway in WNV pathogenesis remains elusive. We investigated the role of PI3K in WNV pathogenesis and immune responses in primary mouse macrophages which constitute an important fraction of the inflammatory infiltrate seen in the central nervous system of WNV patients^19^, primary embryonic fibroblasts and two human cell lines. We found that PI3K inhibitors significantly enhanced WNV replication, and simultaneously repressed IFN-I protein production. Furthermore, we demonstrated that the inhibitor of the catalytic subunit p110δ of class I PI3K decreased IFN-I significantly. Lastly translocation of interferon regulatory factor 7(IRF7) from the cytosol to the nuclei was effectively blocked in the presence of PI3K inhibitors. Our results show that PI3K signaling is critical for the control of WNV infection by modulating IFN-I response.

## Results

### PI3K signaling is critical for the control of West Nile virus infection by modulating type I IFNs response

**Figure 1:**
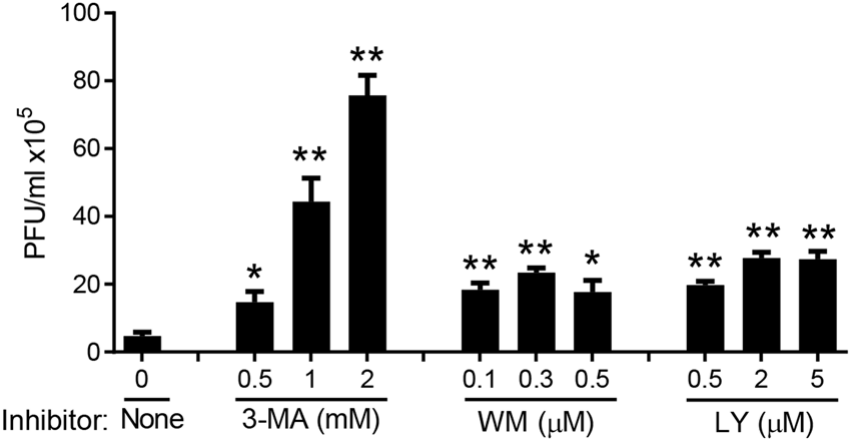
Inhibition of PI3K enhances WNV infection of bone marrow derived macrophages (BMDMs). BMDMs were infected with WNV in the presence of DMSO (None), or various concentrations of 3-methyadenine (3-MA), Wortmannin (WM) and LY294002 (LY) for 24 h. The numbers of viral particles in the cell culture media are expressed as plaque forming units (PFU) per milliliter. *p < 0.05, **p < 0.001 (two-tailed unpaired Student’s T-test), Bar: the mean of the result + s.e.m. The data shown are representative of 3 independent reproducible experiments.

**Figure 2:**
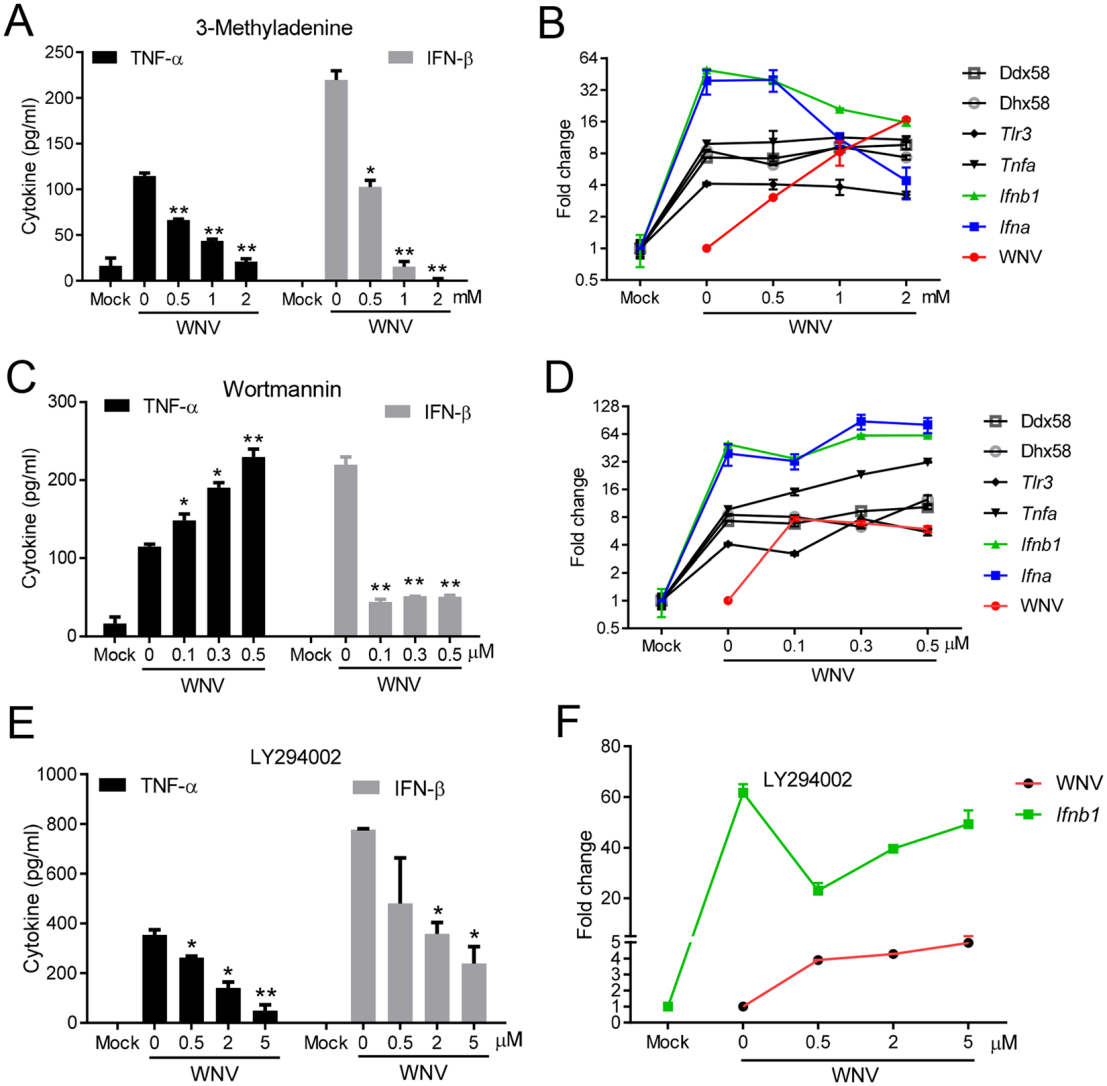
Inhibition of PI3K impairs WNV-induced immune responses in macrophages. BMDMs were left untreated (mock) or infected with WNV in the presence of various concentrations of (**A**,**B**) 3-methyadenine, (**C**,**D**) Wortmannin and (**E**,**F**) LY294002 for 24 h. (**A**,**C**,**E**) ELISA of TNF-α, IFN-β in the cell culture media. (**B**,**D**,**F**) The cellular viral loads and transcript levels of selected genes were quantified by q-PCR. The results of immune gene expression are expressed as fold induction in WNV infected cells over mock. The viral loads are expressed as fold change in inhibitor-treated over untreated cells (0). *p < 0.05, **p < 0.001 (unpaired Student’s T-test), Bar: the mean of the result + s.e.m. The data shown are representative of 3 independent reproducible experiments.

**Figure 3:**
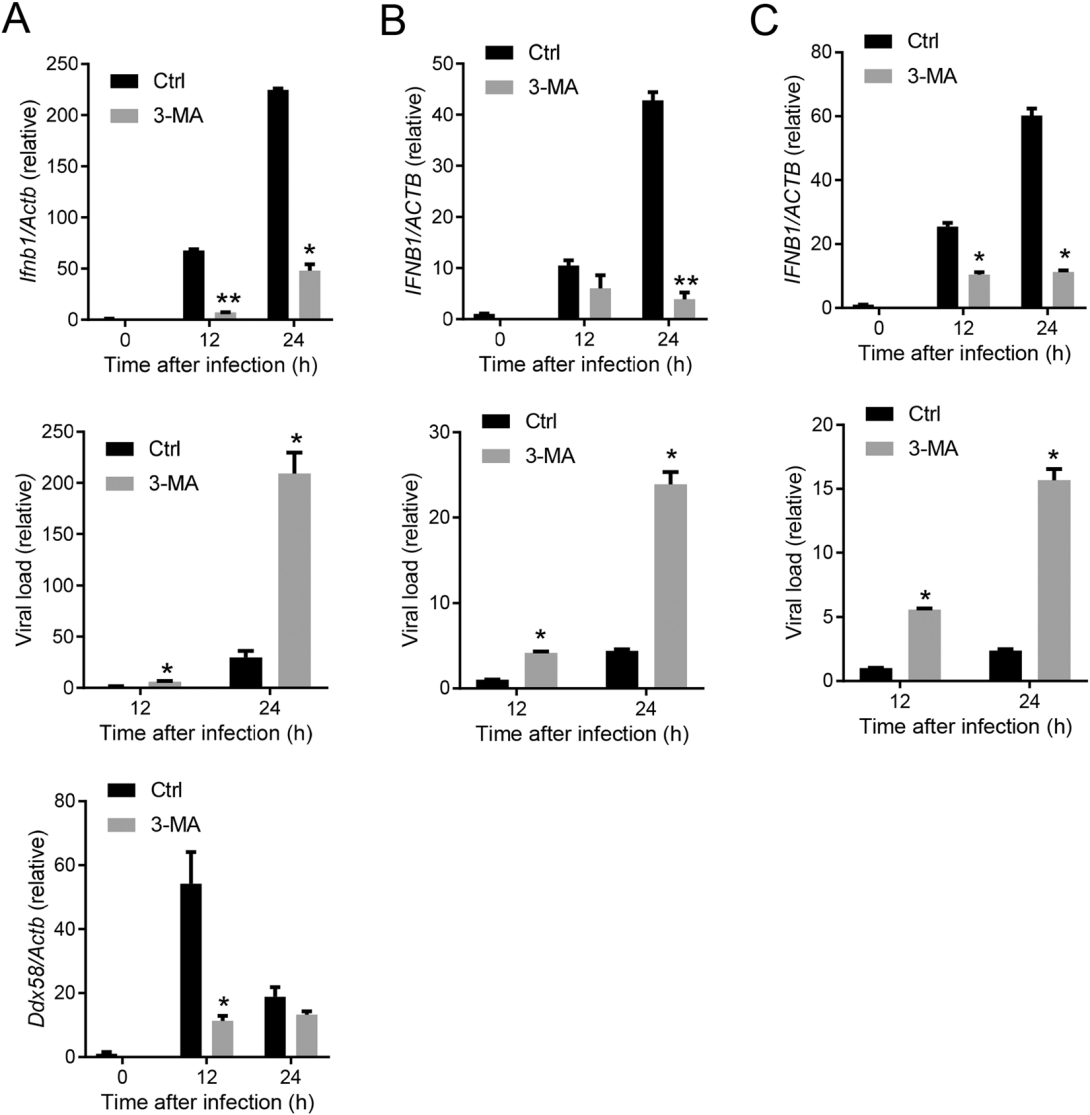
Inhibition of PI3K impairs WNV-induced immune responses in mouse embryonic fibroblasts and human cells. (**A**) MEFs, (**B**) THP-1 and (**C**) H1975 cells were infected with WNV without (Ctrl) or with 2 mM 3-MA for the indicated time. The cellular viral loads and transcript levels of selected immune genes were quantified by q-PCR. The results of immune gene expression are expressed as fold induction in WNV infected cells over mock (0 h). The viral loads are expressed as fold change over 12 h Ctrl cells. Bar: the mean of the result + s.e.m. *p < 0.05, **p < 0.001 (unpaired Student’s T-test). The data shown are representative of 2 independent reproducible experiments.

On one hand, PI3K/Akt signaling is critical for many viruses to complete their life cycle, including cellular entry, replication and egress^16^. On the other hand, the PI3K/Akt pathway also induces antiviral immunity, particularly type I interferon (IFN-I) response^10, 11^. However, the exact role (favorable or unfavorable for virus infection) of the PI3K/Akt pathway may be virus-specific. We tested West Nile virus (WNV), a reemerging flavivirus of significant public health concern in the United States. Treatment of cells with a PI3K inhibitor, 3-methyladenine (3-MA), enhanced WNV titers by 3–16 folds in a dose dependent manner (Fig. 1). Two other pan-PI3K inhibitors, Wortmannin (WM) and LY294002 (LY) also increased viral infection by more than 4 times. These data clearly demonstrate that the PI3K/Akt pathway is essential for prevention of WNV infection. We next asked how PI3K controls WNV infection. PI3K may prevent viral infection by inducing cell-intrinsic antiviral mechanisms such as IFN-I response^18^ and autophagy^16^. Indeed, both secreted TNF-α and IFN-β protein concentrations in the cell culture medium decreased dramatically after 3-MA treatment in a dose dependent manner (Fig. 2A). At the mRNA level, *Ifna* was most dramatically repressed by 3-MA treatment (4 and10 times at 1 mM and 2 mM respectively); *Ifnb1* was down-regulated modestly by 2–3 times. However, *Tnfa, Tlr3, Ddx58 and Dhx58* mRNA expression was not significantly influenced by 3-MA at any concentrations (Fig. 2B). In the case of Wortmannin treatment, IFN-β protein concentration was reduced by 4 times (Fig. 2C), but *Ifnb1*, *Ifna* and *Tlr3* mRNA expression was up-regulated slightly; *Ddx58* and *Dhx58* remained unchanged (Fig. 2D). Surprisingly, TNF-α protein abundance and mRNA expression was increased in the presence of WM in a dose dependent manner (Fig. 2C,D). Both TNF-α and IFN-β protein concentrations were also reduced significantly by LY294002 treatment, while IFN-β mRNA level was modestly downregulated (Fig. 2E,F). To see if the anti-WNV function of PI3K is universal or cell type-specific, we examined WNV infection with/without 3-MA treatment in primary mouse embryonic fibroblasts (MEFs) and two human cell lines, H1975 (lung epithelial cells) and THP-1 (macrophage-like monocytes). IFN-β mRNA induction by WNV was dramatically repressed in the presence of 3-MA and viral replication was enhanced at 12 and 24 h after infection in all the cell types (Fig. 3A–C). The *Ddx58* mRNA level was 5-fold lower in 3-MA than mock-treated MEFs at 12 h after infection (Fig. 3A). Together, these results suggest that PI3K can inhibit WNV replication through modulating IFN-I response.

### PI3K regulates IRF7 nuclear translocation

**Figure 4:**
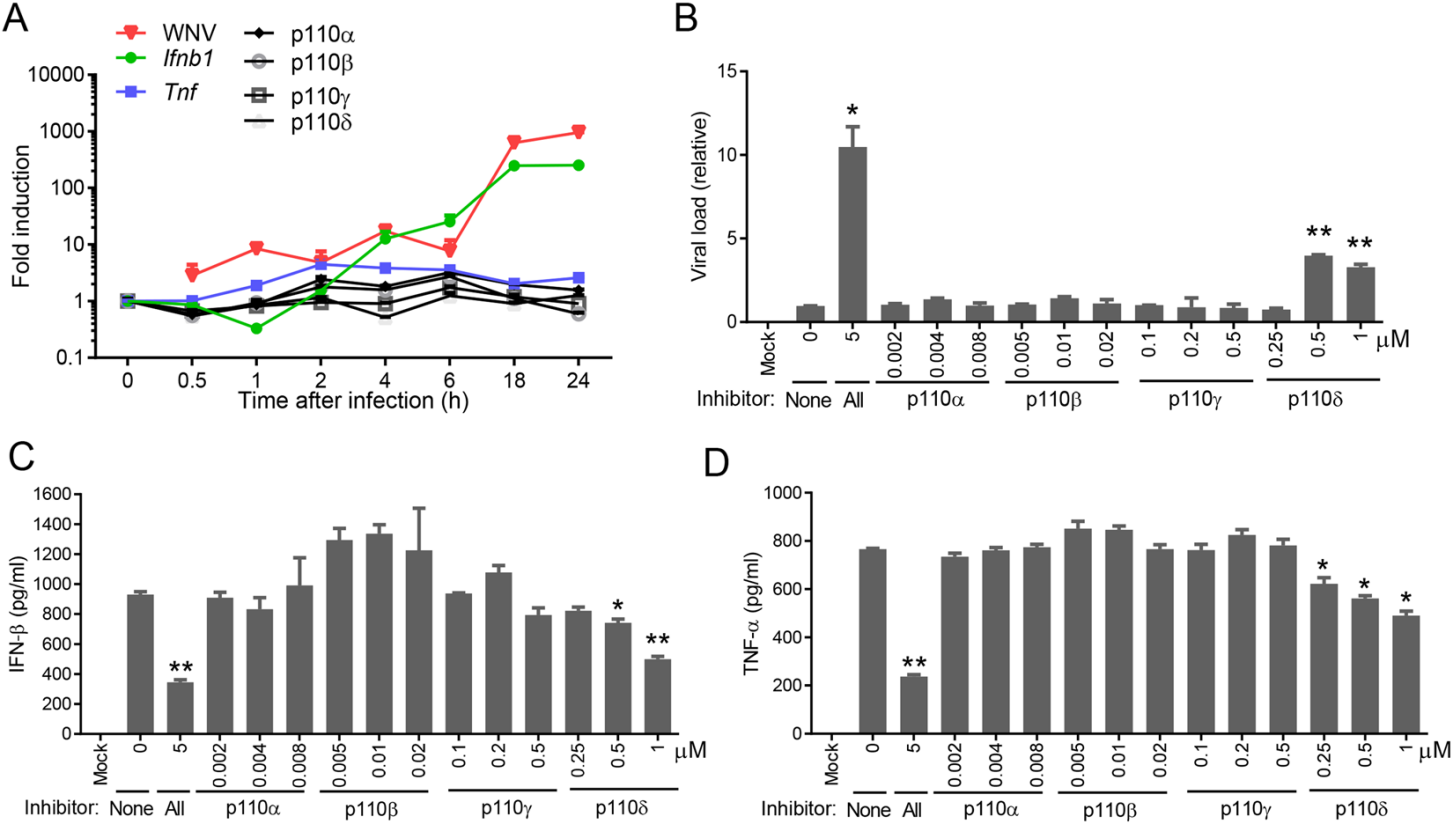
Selective inhibition of the PI3K catalytic p110 δ reduces antiviral immune responses. (**A**) The mRNA abundances of *Ifnb1*, *Tnfa* and p110 isoforms during the course of WNV infection were quantified by q-PCR. The results are expressed as fold change over uninfected (0 h after infection). (**B–D**) BMDMs were left untreated (mock) or infected with WNV in the presence of 5 µM of a pan-PI3K inhibitor LY294002 (All) or various concentrations of p110 isoform-specific inhibitors for 24 h. None: no inhibitor. (**B**) The cellular viral loads were quantified by q-PCR. The data are expressed as fold change over None. ELISA of (**C**) IFN-β and (**D**) TNF-α levels in the cell culture media. *p < 0.05, **p < 0.001 (unpaired Student’s T-test), Bar: the mean of the result + s.e.m. The data shown are representative of 3 independent reproducible experiments.

**Figure 5:**
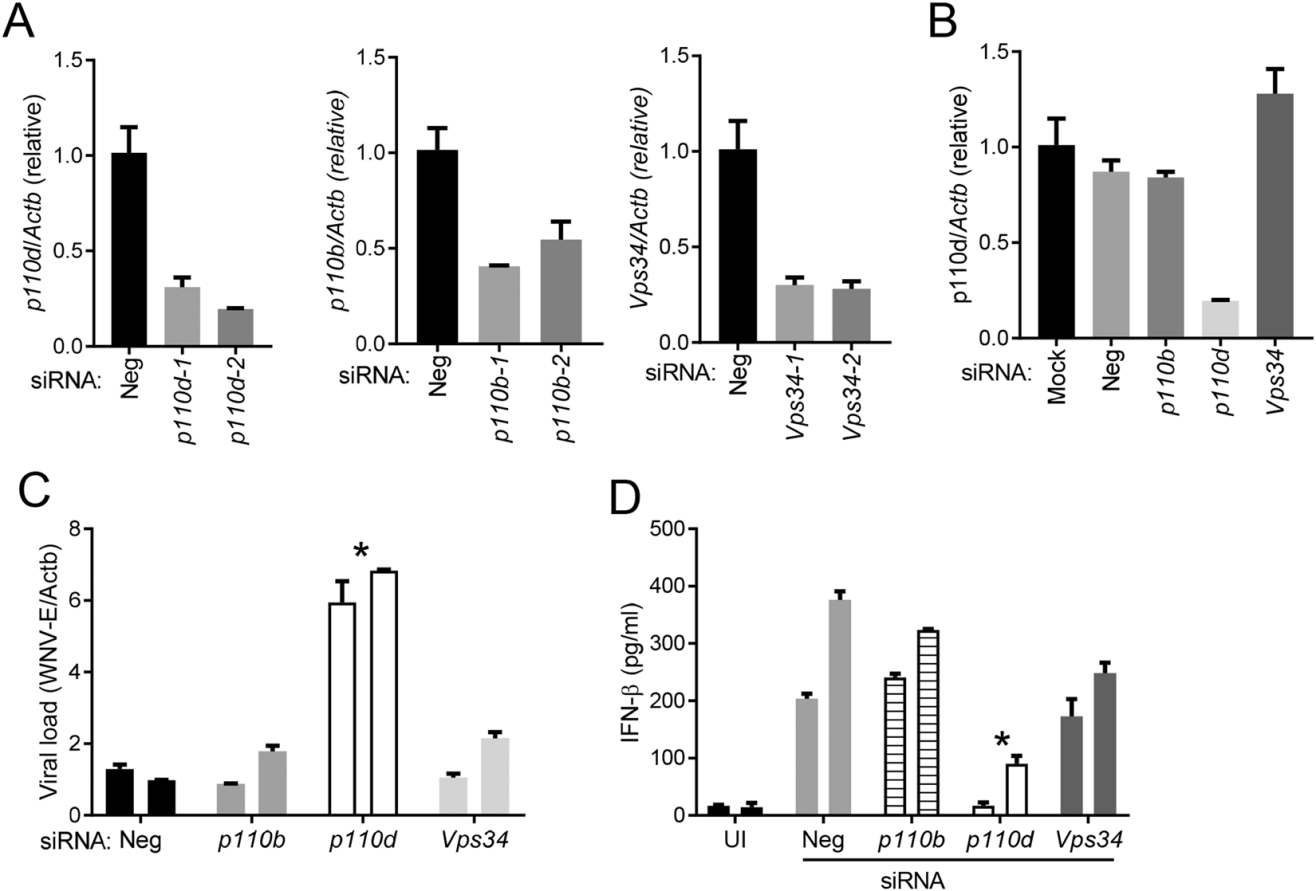
siRNA knock down of the PI3K catalytic p110 δ reduces antiviral immune responses. The scrambled (Neg) or p110-targeting siRNA was electroporated into BMDMs, 48 h later the BMDMs were infected with WNV for 24 h. (**A**,**B**) The mRNA abundances of p110 isoforms were quantified by q-PCR after WNV infection. The results are expressed as fold change over Neg in (**A**) or Mock transfected in (**B**). (**C**) The cellular viral loads were quantified by q-PCR. The data are expressed as fold change over Neg. (**D**) ELISA of IFN-β in the cell culture medium. UI, uninfected. *p < 0.05 (unpaired Student’s T-test), Bar: the mean of the result + s.e.m. The data shown are representative of 3 independent reproducible experiments.

**Figure 6:**
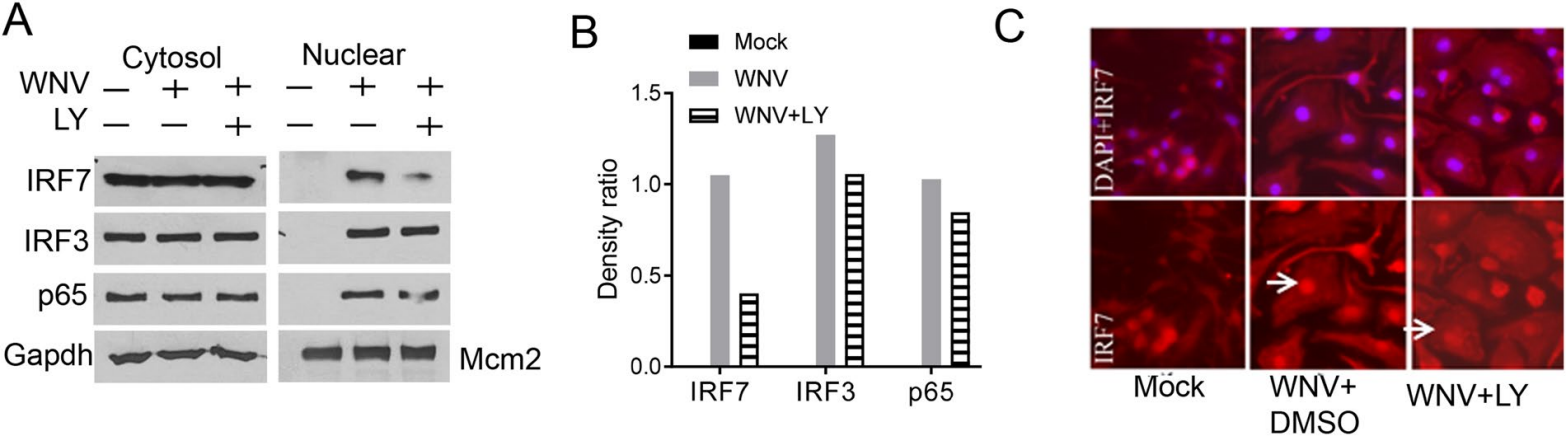
PI3K is critical for WNV-activated nuclear translocation of IRF7. BMDMs were left untreated (mock) or infected with WNV in the presence of vehicle control (DMSO) or 5 μM of LY294002 (LY) for 24 h. (**A**) Immunoblotting analyses of IRF3, IRF7, and NF- κBp65 in the cytoplasmic (Gapdh as the housekeeping protein control) and nuclear fractions (Mcm2 as the housekeeping protein control). Blots shown are cropped from full-length. (**B**) Quantification of the nuclear immunoblot band density in (**A**) by Image J. The results are expressed as the ratio of IRF7/IRF3/p65 to Mcm2. (**C**) Immunofluorescence staining of IRF7 (red) and nuclei by DAPI (blue). Images were acquired with a Zeiss fluorescence microscope. The arrows indicate nuclear IRF7 staining. The data shown are representative of 3 independent reproducible experiments.

The family of PI3Ks is grouped into 4 classes, with class I being extensively studied^16^. The class I PI3K catalytic p110 subunit has 4 isoforms: α, β, γ and δ. To determine which isoform was important for IFN-Is induction after WNV infection, we examined the mRNA levels of all the four p110 subunits. Accompanied by increasing viral replication and immune response (*Infb1* and *Tnfa*), p110α and p110β mRNA expression increased by 2–3 fold between 2–6 h after infection; while p110γ and p110δ remained unchanged during the course of infection (Fig. 4A). However, only p110δ inhibitor enhanced WNV replication by 4-fold and decreased TNF-α by 1.56 and IFN-β production by 1.8 fold at 1 µM, to a lesser extent than the pan-PI3K inhibitor LY290042 though (Fig. 4B–D). To validate the pharmacological results, we next used siRNA to knock down p110δ expression. We used two unique siRNA against p110β, p110δ and Vps34, all of which achieved >60% silencing efficiency (Fig. 5A). The p110β and Vps34 siRNA did not influence p110δ expression, suggesting specificity of the silencing effect (Fig. 5B). Consistent with the pharmacological inhibition (Fig. 4), p110δ knockdown enhanced viral replication by ~6 fold (Fig. 5C) and simultaneously decreased IFN-β production by 4–11 times (Fig. 5D), compared to the negative (scrambled siRNA) controls. These results suggest that class I p110δ is the dominant catalytic subunit for IFN-I induction by WNV. To further investigate the mechanism of interferon induction by PI3K, we examined three transcription factors critical for IFN-I expression, interferon regulatory factors (IRF) 3,−7 and NF-κB. We noted that WNV infection increased the protein amounts of IRF3, −7 and NF-κB in the nuclear fraction when compared to non-infected nuclear extracts. The amounts of nuclear IRF7 were reduced by 62% in the presence of LY294002, while IRF3 and NF-kB p65 was modestly decreased by 17% (Fig. 6A,B). We confirmed IRF7 nuclear translocation deficiency by immunofluorescence microscopy: the intensity of nuclear IRF7 was lower in LY294002-treated WNV-infected cells than DMSO-treated WNV-infected cells (Fig. 6C).

## Discussion

The PI3K/Akt pathway regulates a variety of cellular processes, including cell proliferation, RNA processing, protein translation, autophagy and apoptosis^6^. In order to replicate in host cells, viruses develop strategies to stimulate basal PI3K activity^16^. As an adaptive strategy, PI3K may activate IFN-I response and autophagy to counteract viral invasion. However, autophagy may be hijacked by viruses for their efficient replication, likely in a cell-type and virus specific manner ^20^. Autophagy is induced by WNV infection in many cell types including fibroblasts^21^, neurons^22^ and macrophages (our unpublished data); its role in WNV replication however is still controversial. One study showed that autophagy inhibited WNV replication in mouse embryonic fibroblasts infected with a low multiplicity of infection (MOI = 0.01)^21^. While other studies demonstrated that autophagy played no role in WNV replication in cell lines derived from kidney, liver, skin and^23^, and in IFN-I-deficient Vero/BHK and primary neuron cultures^22^. Inhibition of PI3K with 3-MA repressed WNV replication in primary neurons^22^, suggesting PI3K signaling favors viral replication. In contrast to this report, our study shows that PI3K is critical for control of WNV replication and type I IFN response in primary macrophage, an important immune cell type that restricts WNV infection *in vivo* ^24^ and several other cell types. Macrophages may control WNV infection by direct effector mechanisms such as the production of nitric oxide intermediates^25^ or production of IFN-I^26^. Our study indicates that PI3K positively regulates IFN-β protein expression.

Furthermore, we found that the modes of action of three pan-PI3K inhibitors on type I IFNs are different. 3-MA and LY294002 interfere with IFN-I expression at both the transcriptional and translational level. Interestingly, although 3-MA reduces the TNF-α protein concentration in the culture media dramatically, it has no effect on TNF-α mRNA expression. A possible explanation may be that 3-MA interferes with TNF-α translation or secretion. In macrophages, TNF-α is transported to the cell surface where it is cleaved by TNF converting enzyme and released into extracellular milieu via constitutive exocytosis and recycling endosomes^27–29^ involving PI3Ks. Wortmannin does not influence type I IFN transcription significantly, but reduces protein abundance in the culture media. It is plausible that Wortmannin preferentially targets PI3Ks that are involved in IFN-I secretion, which underlying mechanism is currently unknown. Wortmannin may also inhibit other kinases such as myosin light chain kinase (MLCK) and phosphatidylinositol 4-OH kinase (PI4K) that potentially play a role in exocytosis^30, 31^. Surprisingly Wortmannin enhances both TNF-α protein and mRNA expression, which may be a secondary result of increased viral replication. Our results suggest that Wortmannin selectively interferes with IFN-I response and is a helpful tool for study of the molecular pathway governing type I IFN secretion.

Lastly, we identify the catalytic isoform of class I PI3K required for IFN-I induction. Inhibition of p110δ, but not other isoforms down-regulates type I IFN expression. However, the class I p110δ inhibitor is less potent than the pan-PI3K inhibitors to repress WNV-induced IFN-I expression, suggesting that other classes of PI3Ks also play a role. Consistent with our findings, it was recently shown that p110δ is essential for IFN-α production by plasmacytoid dendritic cells (pDCs) in response to TLR stimulation^32^.

In summary, our results clearly define an antiviral role for PI3K by modulating IFN-I immune responses and demonstrate differential mode of action of three PI3K inhibitors on IFN-I. Future work is to understand the molecular pathways governing IFN-I secretion using Wortmannin.

## Materials and Methods

### Cell culture and viral infection

All animal protocols were approved by the Institutional Animal Care & Use Committee at New York Medical College adhering to the National institutes of Health recommendations for the care and use of laboratory animals. Bone marrow derived macrophages (BMDMs) were differentiated as described previously^33^. Briefly, Bone marrows were isolated from the tibia and femur bones of 6 weeks old female C6/57BL (The Jackson Laboratory, # 000664. https://www.jax.org/strain/000664) and then differentiated into macrophages (BMDMs) in L929 (ATCC, #CCL-1) conditioned medium (RPMI1640, 20%FBS, 30% L929 culture medium, 1x antibiotics/antimycotics) in 10-cm Petri-dishes at 37 °C for 5 days. The attached BMDMs were dislodged by pipetting and counted for plating. BMDMs were cultured in Roswell Park Memorial Institute (RPMI) 1640 medium, Vero cells in DMEM, containing 10% (volume/volume) fetal bovine serum (FBS, Invitrogen, Carlsbad, CA), 100U/ml penicillin and 100 μg/ml streptomycin (Invitrogen) and maintained at 37 °C and 5% CO_2_. The WNV strain was a CT2741 isolate of NY1999^34^ and propagated in C6/36 cells to generate a stock of ~1 × 10^7^ PFU/ml^35^. After differentiation, BMDMs were cultured in RPMI medium overnight, washed once with prewarmed fresh medium and then infected with WNV at a multiplicity of infection (MOI) of 10. After 1 hr at 37 °C, the inoculum was replaced with fresh culture medium and cells were incubated at 37 °C. Cells and culture supernatants were collected at the indicated time points for further analyses.

For preparation of mouse embryonic fibroblasts (MEFs), day 14 embryos were decapitated and eviscerated then digested with trypsin for 10 min at 37 °C rotating. Fibroblasts were filtered through a 100 μM filter, cultured in RPMI1640 medium (Life Technologies, NY 14072 USA) supplemented with 10% fetal bovine serum and antibiotics/antimycotics, propagated for 2 passages and then frozen. MEFs were infected with WNV at a MOI of 1.

H1975 (human lung epithelial cell, Cat# CR5908) and THP-1 (human monocytes, Cat# TIB202) were purchased from American Type Culture Collection (ATCC) (Manassas, VA20110, USA). These cells were cultured in RPMI1640 medium and infected with WNV at a MOI of 1 and 5 respectively.

### Reagents and Antibodies

3-Methyladenine (IC50 = 4.5 mM), Wortmannin (IC50 = 3 nM) and IC-87114 (PI3K subunit p110δ, IC50 = 0.5 µM)) were purchased from Cayman Chemical (Ann Arbor, Michigan 48108, USA). LY294002 (IC50 = 1.0 µM), another PI3K inhibitor, was obtained from the Invivogen (San Diego, CA 92121, USA). The PI3-Kinase p110 α subunit inhibitor (B-0304, IC50 = 2 nM)), and γ inhibitor B-0302(IC50 = 250 nM) were from the Echelon (Salt Lake City, UT 84108, USA). The selective inhibitor of the P13K subunit p110 β, TGX-221(IC50 = 5 nM) was from EMD Millipore (Billerica, Massachusetts 01821, USA). Rabbit anti-IRF-7 (H-246) was purchased from Santa Cruz Biotechnology (Dallas, TX 75220 USA). Rabbit anti-GAPDH (Cat #5174), IRF-3 (Cat # 4302) and NF-κB p65 (Cat #4764) antibodies were available from Cell Signaling Technology (Danvers, MA 01923 USA). Goat anti-MCM2 (Cat # A300-122) was from Bethyl Laboratories (Montgomery, TX 77356, USA). The siRNA oligonucleotides were purchased from Ambion of Thermo Fisher Scientific. The sequences are p110b (ID: s93108, 5′-CAUAGAUUUUGGGCAUAUUTT-3′; ID: s93107, 5′-GCAUAGCUGGUCUUCGUUUTT-3′), p110d (ID: s71605, 5′-CGAUGAAGCUGGUUGUUCATT-3′; ID: s71606, 5′-GCCGAAAAGUGAAUGCUGATT-3′), and Vps34 (ID: 165687, 5′-GCUUGCUCGGAGCUUAAGATT-3′; ID: 165688, 5′-CGAAGCUCUAAUUUCAUGUTT-3′).

### Plaque forming assay

The detailed procedures of plaque forming assay were described previously^36^. Briefly, serially diluted culture supernatants from infected cells were inoculated into monolayers of type I IFN deficient Vero cells (ATCC # CCL-81). After 1 h of incubation at 37 °C, the inoculums were removed, and overlay medium (DMEM containing 10% FBS and 1% Seaplaque agarose) was added and incubated at 37 °C for 4 days. Plaques were visualized after Neutral Red (Sigma-Aldrich) staining on a white transilluminator.

### ELISA

Commercial enzyme-linked immunosorbent assay (ELISA) kit were used to measure levels of secreted TNF-α (Cat # 88-7324, Affimetrix, San Diego, CA 92121USA) and INF-β (Cat# 42400, PBL Assay Sciences, Piscataway Township, NJ 08854 USA) proteins in the cell culture supernatants. The procedures were exactly the same as described in the product manuals.

### Quantitative RT-PCR

RNA was extracted following the QIAGEN manual exactly, reverse-transcribed into cDNA using the BIO-RAD iScript™ cDNA Synthesis Kit. qPCR was performed with gene-specific primers and 6FAM-TAMRA (6-carboxyfluorescein–N,N,N,N-tetramethyl-6-carboxyrhodamine) probes, or primers (See Table 1) and SYBR-Green master mix on a BIO-RAD CFX96 Touch™ Real-Time PCR Detection System^35^. The PCR conditions were 50 °C for 2 min, 95 °C for 10 min, 40 cycles of 95 °C for 15 s, and 60 °C for 30 s. Results were calculated using the −ΔΔCt method and beta actin gene as an internal control. Primers and probes are listed in Table 1. The following primer/probe (6FAM-MGB) premixes were obtained from Applied Biosystems of Thermo Fisher Scientific: p110d, Mm00435674_m1; p110b, Mm00659576_m1; p110g, Mm00445038_m1; p110a, Mm00435669_m1; Vps34, Mm00619489_m1; Tlr3, Mm01207404_m1; Ddx58, Mm01216853_m1; Dhx58, Mm01302252_m1.

**Table 1:** Primers and probes.

Gene symbol	Forward 5′-3′	Reverse 5′-3′	Probe 5′-6FAM, 3′-TAMRA
*Mm Actb*	AGAGGGAAATCGTGCGTGAC	CAATAGTGATGATGACCTGGCCGT	CACTGCCGCATCCTCTTCCTCCC
*Mm Ifna*	CTTCCACAGGATCACTGTGTACCT	TTCTGCTCTGACCACCTCCC	AGAGAGAAGAAACACAGCCCCTGTGCC
*Mm Ifnb1*	CTGGAGCAGCTGAATGGAAAG	CTTCTCCGTCAT CTCCATAGGG	CAACCTCACCTACAGGGCGGACTTCAAG
*MmTnfa*	CTCCAGGCGGTGCCTATGT	GAAGAGCGTGGTGGCCC	CAGCCTCTTCTCATTCCTGCTTGTGGC
*WNV-E*	TTCTCGAAGGCGACAGCTG	CCGCCTCCATATTCATCATC	ATGTCTAAGGACAAGCCTACCATC

### siRNA transfection and infection

BMDMs were transfected with siRNA and a mouse macrophage transfection Kit (Lonza #VPA-1009) on a Nucleofector 2. Briefly, differentiated BMDMs were dislodged gently from Petri dishes and counted using trypan blue staining. 1 × 10^6^ cells were centrifuged at 200 × g for 10 minutes at room temperature. The resultant supernatant was discarded completely and the cell pellet was washed once with 1 ml sterile PBS. PBS was completely removed. The cell pellet was re-suspended carefully in 100 µl room temperature Nucleofector Solution VPA-1009 and 200 pmol siRNA per sample. The mix was transferred into a certified cuvette (sample must cover the bottom of the cuvette without air bubbles) and electroporated with a Nucleofector II using program Y-001. ~500 µl of the pre-equilibrated culture medium was added to the cuvette and then gently transferred to a 12-well cell culture plate. 48 h after transfection, the cells were infected with WNV for 24 h.

### Nuclear extraction

5 × 10^6^ cells were scraped off from culture dishes and washed with ice cold phosphate buffered saline (PBS) twice. The cells were then resuspended gently in 500 μl 1x Hypotonic Buffer (20 mM Tris-HCl, pH 7.4, 10 mM NaCl, 3 mM MgCl_2_, plus protease inhibitors) by pipetting up and down several times and then incubated on ice for 15 minutes. 25 μl detergent (10% NP40) was added to the cell suspension, which was then vortexed for 10 seconds at highest setting. The homogenate was centrifuged for 10 minutes at 3,000 rpm at 4 °C, resulting in supernatant (cytosol) and pellet (nuclei). The nuclei were lyzed in 50 μl complete Cell Extraction Buffer (10 mM Tris, pH 7.4, 2 mM Na_3_VO_4_, 100 mM NaCl, 1% Triton X-100, 1 mM EDTA, 10% glycerol

1 mM EGTA, 0.1% SDS, 1 mM NaF, 0.5% deoxycholate, 20 mM Na4P_2_O_7_, complete protease inhibitors) for 30 minutes on ice with intermittent vortexing every 10 minute. The lysate was then centrifuged for 30 minutes at 14,000 × g at 4 °C, resulting in nuclear lysate (supernatant).

### Western blotting

For immunoblotting, cells were harvested at indicated time points after WNV infection, lysed in lysis buffer buffer [50 mM Tris–HCl (pH 7.5), 150 mM NaCl, 5 mM EDTA, 1% Triton X-100] supplemented with Complete Protease Inhibitor Cocktail (Roche Diagnostics, Indianapolis, IN). Cell lysates were resolved on a 4- to 15%-gradient SDS-PAGE gel, and the separated proteins were transferred to a nitrocellulose membrane. The proteins of interest on the membrane were probed with specific antibodies, followed by HRP-conjugated secondary antiserum, and detected with an enhanced chemilumiscence system from GE Healthcare (Port Washington, NY 11050 USA).

### Immunofluorescence microscopy

For immunofluorescence assay, macrophages were fixed with 4% paraformaldehyde (PFA) in phosphate buffered saline for 10 min. The cells were sequentially permeabilized with 0.5% Triton X-100, blocked with 2% goat serum, incubated with primary antibody (rabbit anti-IRF7 at 1:100 dilution,) at 4 °C overnight, incubated with Alexa Fluor 488-conjugated goat anti-rabbit IgGs (Life Technologies Cat #A11070) for 1 h at room temperature. Nuclei were counter stained with DAPI. Images were acquired using a Zeiss fluorescence microscope with a 40x objective (Oberkochen, Germany).

### Graphing and statistics

Prism 7 software (GraphPad Software) was used for charts and statistical analyses. A standard two-tailed unpaired Student’s t-test was used to calculate p values, which were considered significant if ≤ 0.05. The sample sizes (biological replicates), specific statistical tests used, and the main effects of our statistical analyses for each experiment were detailed in each figure legend.

### Data Availability

The datasets generated during and/or analyzed during the current study are available from the corresponding author on reasonable request.
